# Stroke prevention in patients with atrial fibrillation and comorbidities: evidence and common sense

**DOI:** 10.1007/s12471-020-01491-1

**Published:** 2020-09-08

**Authors:** J. R. de Groot

**Affiliations:** Department of Cardiology, Heart Center, Amsterdam UMC, University of Amsterdam, Amsterdam Cardiovascular Sciences, Amsterdam, The Netherlands

Patients with atrial fibrillation (AF) are at increased risk of thromboembolic stroke; therefore, the use of oral anticoagulation is recommended for all patients with a CHA_2_DS_2_VASc score of 2 or higher (3 or higher for females). Oral anticoagulation should also be considered for patients with a CHA_2_DS_2_VASc score of 1 (2 for females) [1]. Indeed, this last group is at a considerably increased risk of stroke as well, indicating that oral anticoagulation is often required, unless there is a good clinical reason to abstain [2]. Possible reasons to reconsider the need for anticoagulation include a perceived high bleeding risk—an increased bleeding risk calls for addressing the risk factors for bleeding, rather than for omitting anticoagulation [1]—or the presence of comorbidities with or without the need for additional antiplatelet therapy.

Historically, vitamin K antagonists (VKAs) were the drug of choice for stroke prevention in AF. A meta-analysis of six randomised clinical trials, including a total of 2900 patients using dose-adjusted warfarin, has demonstrated a risk reduction of 64% compared with placebo [3]. Based on these trials, and in the absence of an alternative, VKAs became the drug of choice for stroke prevention in AF across a wide range of patient populations for several decades.

With the publication of four large phase 3 trials on the efficacy and safety of non-vitamin K antagonist oral anticoagulants (NOACs, also referred to as direct-acting oral anticoagulants or DOACs), consisting of the thrombin inhibitor dabigatran and the factor Xa inhibitors rivaroxaban, apixaban and edoxaban, a large body of evidence on stroke prevention in AF became available [4–7]. In a meta-analysis of more than 70,000 participants in these randomised studies, DOACs proved to be significantly more efficacious than VKAs, with a 19% reduction in stroke or systemic embolism and a 10% reduction in all-cause mortality compared with warfarin. Furthermore, major bleeding decreased with 14% compared with warfarin, and intracranial bleeding with 52% [8]. The large number of patients included in these trials allowed for numerous post-hoc subanalyses, which shed light on whether the differential efficacy and safety of DOACs compared with VKAs was still present in patients with comorbidities. Such studies may be criticised for being underpowered: the selected populations may not fully reflect clinical reality and the studies are primarily hypothesis generating. Still, one should take into consideration that, for example, the number of patients in the subgroup >75 years of age in the NOAC trials alone exceeds the *total* number of participants in the VKA trials with more than a factor of 8 [9].

However, conditions and situations that have not been addressed in randomised NOAC trials remain, particularly with respect to comorbid disease or the need for concomitant use of medication affecting the thrombosis or bleeding risk. This issue of the *Netherlands Heart Journal* features a report by Mulder et al. of a multidisciplinary advisory meeting on decision-making on NOAC use in complex clinical situations that took place in June 2019 [10]. The authors focus on four specific situations. In AF patients who have undergone percutaneous coronary intervention (PCI), the concomitant use of oral anticoagulation and antiplatelet therapy is indicated to prevent stent thrombosis. However, adding antiplatelets, especially dual antiplatelet therapy, to oral anticoagulation (VKA or DOAC) significantly increases the risk of bleeding, while omitting antiplatelets results in an unacceptable risk of stent thrombosis.

The open-label WOEST trial already showed in 2011 that dual therapy, consisting of a VKA and clopidogrel, is associated with a significant reduction in bleeding complications compared with triple therapy (VKA plus aspirin plus clopidogrel), without evidence of increased thrombotic risk [11]. Following the four randomised trials in AF patients undergoing PCI [4–7], triple therapy (oral anticoagulant plus aspirin plus P2Y_12_ inhibitor) should be prescribed for as short a time period as possible, and the use of dual therapy should be restricted to 6 to 12 months, depending on the bleeding risk of the individual patient [12–15]. Of note, a meta-analysis of the four DOAC PCI trials has demonstrated a numerically small increase in stent thrombosis in patients using a DOAC plus single antiplatelet therapy compared with patients who used a VKA plus double antiplatelet therapy (56 vs 30 cases, risk ratio 1.55, 95% confidence interval 0.99–2.41), which was counterbalanced by a 38% lower bleeding risk in the DOAC groups (634 vs 804 cases) [15]. Hence, the duration of antiplatelet therapy needs to be limited to mitigate the bleeding risk. There is no evidence for off-label reduction of the DOAC dose.

In AF patients with peripheral artery disease, in the absence of recent stenting, single therapy with a DOAC without the addition of antiplatelets appears sufficient in most cases, but the authors suggest that in highly symptomatic patients addition of an antiplatelet drug to the full DOAC dose may be considered, although solid evidence supporting this advice is lacking [10].

Ischaemic or haemorrhagic stroke in AF patients requires temporary discontinuation of DOAC therapy, to prevent (further) haemorrhagic deterioration and to allow thrombolysis when possible. The European Heart Rhythm Association’s consensus document provides guidelines on when to reintroduce anticoagulation following an ischaemic stroke or intracranial bleeding. In general, and related to the size of the ischaemic stroke, the advised time to restart the DOAC varies between ≥1 day following a transient ischaemic attack and 12–14 days after a large ischaemic stroke with persisting neurological deficits [16]. Of note, the ANNEXA‑4 study has investigated the factor Xa inhibitor antidote andexanet alfa in 352 patients with predominantly major intracranial (64%) or gastrointestinal (26%) bleedings [17]. In this study, 10% of patients experienced thromboembolic events and 14% died within 30 days after the bleeding, mostly before restart of anticoagulation. This indicates that, although life-threatening bleeding is a severe concern in patients using DOACs, their thromboembolic risk remains unchanged. Restarting the DOAC after a bleeding is crucial, albeit that the timing of the restart is not as well defined for haemorrhagic stroke as it is for ischaemic stroke.

The last clinical scenario Mulder et al. touch upon is the patient with an active malignancy. Particularly in case of a gastrointestinal or urogenital tumour, there is concern about the use of DOACs. Also, there may be an interaction between several cancer treatments and the bleeding risk may be further attenuated by thrombocytopenia. Subanalyses of the rivaroxaban and edoxaban trials have indicated that the benefit of factor Xa inhibitors, compared with VKAs, is maintained in patients with active cancer.

How should we approach these or other complex clinical pathologies in our daily clinical practice? Several decisions need to be taken. First, it has to be decided whether the AF patient with comorbid disease requires anticoagulation. Second, a choice needs to be made between a DOAC and a VKA and at which (reduced) dose. Third, the use of concomitant medication needs to be assessed. Mulder et al. summarise their findings by recommending to prescribe a DOAC to the AF patient with comorbidities, principally at the dose studied in the randomised trials. The role of VKAs for stroke prevention in AF is marginal and, with the availability of more randomised and observational data on DOACs, continues to move further to the periphery of indications. The concomitant use of antiplatelet drugs in AF patients with a coronary or peripheral arterial stent needs to be continued for as short a period as reasonably possible.
